# Association Between MTHFR C677T Polymorphism and Susceptibility to Autism Spectrum Disorders: A Meta-Analysis in Chinese Han Population

**DOI:** 10.3389/fped.2021.598805

**Published:** 2021-03-10

**Authors:** Chen-Xi Li, Yi-Guang Liu, Yue-Ping Che, Jian-Lin Ou, Wen-Cong Ruan, Yong-Lin Yu, Hai-Feng Li

**Affiliations:** ^1^Department of Rehabilitation, the Children's Hospital, Zhejiang University School of Medicine, National Clinical Research Center for Child Health, Hangzhou, China; ^2^Department of Linguistics, Zhejiang University, Hangzhou, China; ^3^Department of Rehabilitation Medicine, the First Affiliated Hospital of Jinan University, Guangzhou, China

**Keywords:** autism spectrum disorders, susceptibility, MTHFR, polymorphism, meta-analysis

## Abstract

Prior studies have examined the influence of MTHFR C677T on autism susceptibility, however, there are no consensus conclusions and specific analyses of a Chinese population. This meta-analysis included a false-positive report probability (FPRP) test to comprehensively evaluate the association of MTHFR C677T polymorphism with autism susceptibility among a Chinese Han population. A large-scale literature retrieval was conducted using various databases including PubMed, Embase, Wan Fang, and the Chinese National Knowledge Infrastructure (CNKI) up to July 31, 2020, with a total of 2,258 cases and 2,073 controls included. The strength of correlation was assessed by odds ratios (ORs) and 95% confidence intervals (95% CIs). MTHFR C677T showed a significant correlation with increased ASD susceptibility under all genetic models (T vs. C, OR = 1.89, 95% CI 1.28 to 2.79; TT vs. CC: OR = 2.44, 95% CI 1.43 to 4.15; CT vs. CC, OR = 1.73; 95% CI 1.19 to 2.51; CT + TT vs. CC: OR = 2.03, 95% CI 1.31 to 3.15; TT vs. CT + CC, OR = 1.95, 95% CI 1.21 to 3.13). Stratification analysis by region also revealed a consistent association in the Northern Han subgroup, but not in the Southern Han subgroup. Pooled minor allele frequency (MAF) of 30 studies were 45% in Northern Han and 39% in Southern Han. To avoid a possible “false positive report,” we further investigated the significant associations observed in the present meta-analysis using the FPRP test, which consolidated the results. In conclusion, MTHFR C677T polymorphism is associated with the increased risk of autism in China, especially in Northern Han. For those mothers and children who are generally susceptible to autism, prenatal folate and vitamin B12 may reduce the risk that children suffer from autism, especially in Northern Han populations. In the future, more well-designed studies with a larger sample size are expected.

## Introduction

Autism spectrum disorder (ASD) involves a constellation of neurodevelopmental disorders featuring impaired repetitive behaviors and deficits in terms of social communication, which are associated with genetic factors and other causes (1). The prevalence of autism in people under eight years old has increased from about 1/59 in 2014 (2) to about 1/54 in 2016 (3). In China, autistic children occupy about 0.7% of the total population, with a rapid upward trend shown (4). As to the consequences of ASD, not only do child patients suffer from a lower level of living quality but their families also often bear a substantial burden (5). The high heritability index is a genetic component in the etiology of ASD and the related genetic factors have the highest level of complicacy (1). On the one hand, the various symptoms of autism reveal its nature as a complex disease with multi-genetic changes (6); on the other hand, the variability of phenotype among ASDs subgroups indicates the interactions of susceptibility genes with environmental factors (7). Hence, there is an urgent need to identify the etiology or risk factors of ASD.

Methylenetetrahydrofolate reductase (MTHFR) gets involved in the process of converting homocysteine into methionine, with the latter one, as a cofactor, playing a critical role in regulating homocysteine concentration in the blood (8). Homocysteine and oxidative stress are associated with several neuropsychiatric disorders, e.g., autism (9), schizophrenia (10), depression (11), and attention deficit hyperactivity disorder (12), etc. The existing evidence suggests that several DNA sequence variants (genetic polymorphism) are associated with the MTHFR gene, with NM_005957.5(MTHFR): c.665C>T (p.Ala222Val) (C677T in short) drawing most attention as a single nucleotide polymorphism (SNP) (13). MTHFR C677T polymorphism tends to reduce the efficiency of methyl group production with possible adverse downstream effects on gene expression, and impair the efficiency of the one-carbon (C1) metabolic pathway (14).

Previous studies have mainly focused on the influence of the MTHFR C677T on autism susceptibility, but the findings are still inconclusive. For example, (15) reported a correlation between MTHFR C677T polymorphism and a higher susceptibility to ASD, but this is not consistent with the findings of Dos Santos et al. (16). A recent meta-analysis (17) suggested a significant association between them overall and by ethnicity, and thus the MTHFR C677T polymorphism could be used as a diagnostic marker of autism by ethnic background. Given that China is a vast territory characterized by prominent regional differences, it is worth conducting a subgroup analysis of the Chinese population. The present meta-analysis sought to comprehensively evaluate the genetic association of MTHFR C677T polymorphism with autism susceptibility among a Chinese population, with particular attention to the possible differences between those from the north and the south.

## Materials and Methods

### Search Strategy

This study undertook a large-scale literature retrieval based on various databases including PubMed, Embase, Wan Fang, and the Chinese National Knowledge Infrastructure (CNKI) up to July 31, 2020. For minor allele frequency (MAF), the search strategy was performed as follows: (“MTHFR” OR “methylenetetrahydrofolate reductase”) AND (“polymorphism” OR “genotype”) AND (“Chinese Han” OR “China Han”). For the association between MTHFR gene C677T polymorphism and the susceptibility to ASD, the predefined search terms are as follows with no language restriction: (“MTHFR” OR “methylenetetrahydrofolate reductase”) AND (“autism” OR “autism spectrum disorders”) AND (“polymorphism” OR “genotype”). The references listed in relevant primary articles were manually checked to avoid missing any other related articles.

### Eligibility Criteria

For MAF, any human studies with a focus on the prevalence of MTHFR C677T polymorphism and ASD in relation to Chinese Han ethnicity were included, regardless of their sample size and report type. To assess the potential genetic association, the inclusion criteria included: (1) case-control studies with the distribution of MTHFR C677T mutation frequencies in autism and non-autism patients; (2) presenting the accurate genotype or allele frequency; (3) standard diagnostic criteria for autism diagnosis; (4) research on Chinese individuals. The exclusion conditions are as follows: (1) not related to MTHFR polymorphism and autism research; (2) repeated publications; (3) previous meta-analysis, case reports, reviews, editorials, and comments; (4) not human beings model studies.

### Data Extraction

Two co-authors (Li and Liu) extracted the data independently from all included studies for analysis, including the first author's surname, publication year, country, region, control source, sample size, genotype frequency of case and control, diagnostic criteria, genotyping method and HWE for controls. When facing discrepancy, we returned to the original studies in discussion with a third reviewer (Che).

### Statistical Analysis

To measure the strength of correlation between MTHFR C677T polymorphism and autism risk under five genetic models, Odds ratios (ORs), and 95% confidence intervals (95% CIs) were calculated. We also performed a Chi-square test to determine the Hardy-Weinberg equilibrium (HWE) in the control groups, with *P* < 0.05 indicating disequilibrium. Heterogeneity across the studies was evaluated using both Cochran's Q statistic and the *I*^2^ statistic. Specifically, significant heterogeneity was indicated when *P* was less than 0.10 and *I*^2^ was higher than 50% and a random-effects model (the DerSimonian and Laird method) was supposed to be fitted in this case; otherwise, a fixed-effects model using the Mantel-Haenszel was the suitable choice. Furthermore, we conducted sensitivity analysis according to the HWE status of controls, and the subgroup meta-analyses by region were undertaken for particular relationships. We used Begg's funnel plot and Egger's test to detect potential publication bias. The statistical significance of the ORs was determined by Z test, with *P* < 0.05 indicating a significant difference. Stata 14.0 software was used for the above-mentioned statistical analyses.

To determine whether the significant associations (*P* < 0.05) between the MTHFR C677T polymorphism and the risk of ASD were “noteworthy,” we further calculated the FPRP value. Although it is suggested to draw on statistical power to detect an OR of 1.5 with an α-level equal to the observed *P*-value by Wacholder et al. (18), we decided to present the results for OR of 2 as well to make it more stringent.

Based on the estimated probability that the finding may not be a genuine association, only associations with FPRP < 0.2 were deemed noteworthy, as recommended by Wacholder et al. (18).

## Results

### Characteristics of Included Studies

**Figure 1:**
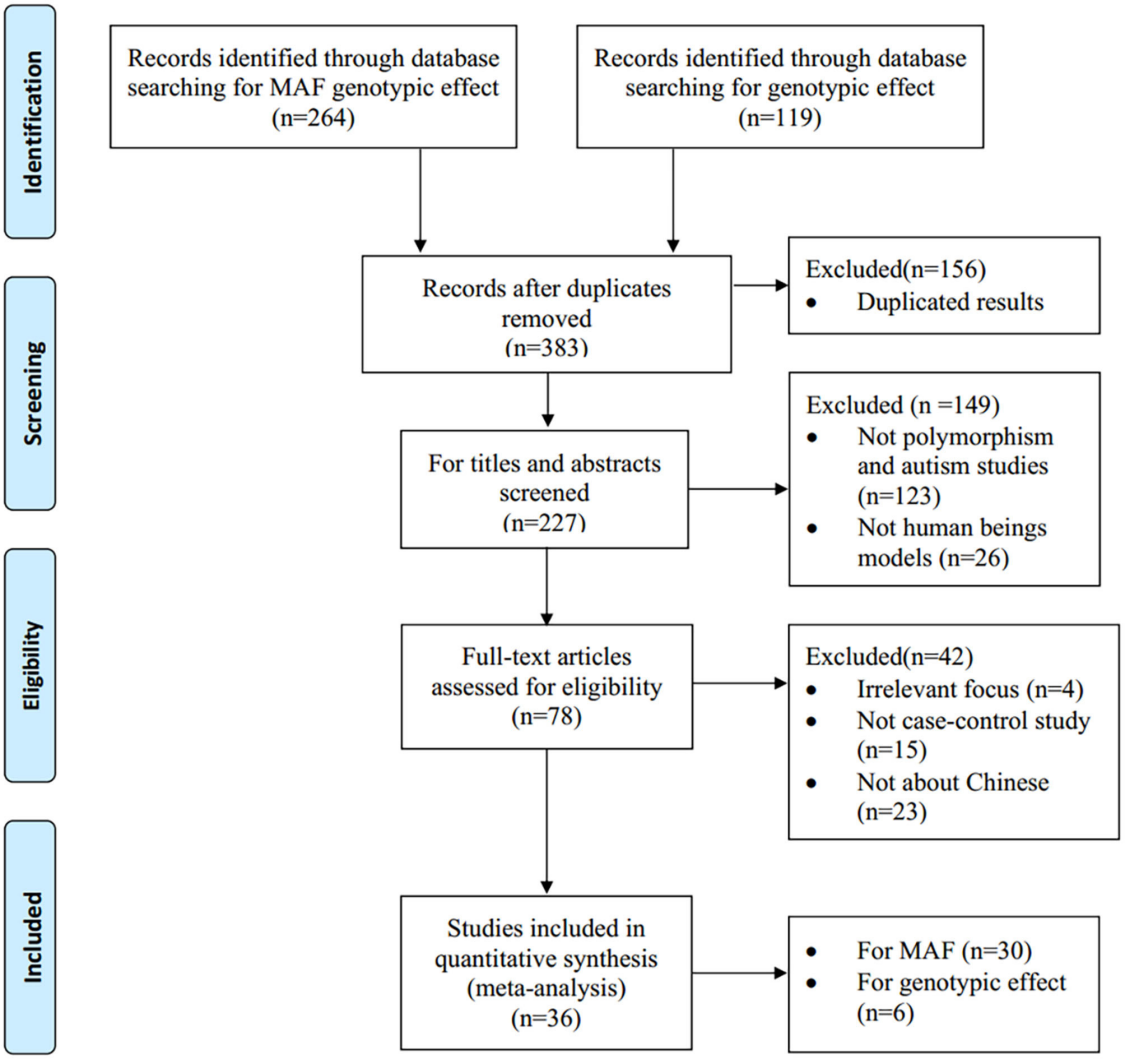
Flow chart of the study selection process.

The procedure of study selection is shown in Figure 1, with the details of the inclusion and exclusion of studies illustrated. For pooling MAF, a total of 264 studies were identified from databases, among which 30 case-control studies were included for pooling minor allele prevalence (19–48), which all reported the MAF in non-ASD populations in Chinese Han. For the targeted gene effect, we yielded 119 papers initially and finally, six publications (49–54) were included in the present meta-analysis, with a total of 2,258 cases and 2,073 controls. Among them, four publications (50–54) concerning Northern Chinese Han, whereas the other two papers (49, 52) focus on Southern Chinese Han. Three studies (50, 51, 54) deviated from HWE. The estimation of MAF is shown in Table 1 and the characteristics of the included studies are summarized in Table 2.

**Table 1:** Estimation of the pooled prevalence of the T allele.

**References**	**P for HWE**	**Total no**.	**T allele frequency(no.)**	**% with T allele**
**Northern**				
Feng et al. (19)	0.308	246	101	41
Li et al. (22)	0.442	640	193	30
Zhang et al. (24)	0.670	2,072	1,141	55
Yang et al. (27)	0.638	1,756	921	52
Fan et al. (28)	0.259	988	516	52
Yun et al. (34)	0.279	416	241	58
Fan et al. (28)	0.662	1,482	787	53
Wang et al. (37)	0.928	336	154	46
Li et al. (40)	0.410	904	186	21
Jiao et al. (39)	0.514	1,098	615	56
Wei et al. (46)	0.323	1,512	683	45
Long et al. (45)^*^	0.021	462	191	41
Wang et al. (48)	0.373	240	57	24
**Southern**				
Xiao et al. (20)	0.521	62	27	44
Li et al. (21)	0.333	290	116	40
Tang et al. (26)^*^	0.045	1,306	582	45
Shen et al. (25)	0.563	438	121	28
Wang et al. (32)	0.259	1,780	740	42
Luo et al. (29)	0.178	270	85	31
Lv et al. (30)	0.374	160	82	51
Lv et al. (31)	0.904	482	190	39
Wang et al. (33)^*^	0.033	1,184	526	44
Hu et al. (36)	0.903	360	146	41
Yuan et al. (38)	0.347	1,024	405	40
Liu et al. (41)	0.657	906	420	46
Wang et al. (44)^*^	0.026	974	430	44
Jiao et al. (42)	0.119	614	283	46
Mao et al. (43)	0.055	396	132	33
Peng et al. (47)	0.070	928	259	30
**Northern and Southern**				
Yang et al. (23)^*^	<0.01	28810	13022	45

* *Not included in pooling minor allele prevalence*.

**Table 2:** Major characteristics of included studies.

**References**	**Country**	**Region**	**Control source**	**Sample size**	**Genotype distribution**						**Diagnostic criteria**	**Genotyping method**	***P* for HWE**
				**Case/control**	**Case**			**Control**					
					**CC**	**CT**	**TT**	**CC**	**CT**	**TT**			
Guo et al. (49)	China	Southern	Healthy	186/186	79	77	30	87	83	16	DSM-IV/ADI-R/ADOS	PCR-RFLP	0.542
Zhaoet al. (50)	China	Northern	Healthy	98/70	52	29	17	60	7	3	DSM- IV	PCR-RFLP	<0.01
Zhaoet al. (51)	China	Northern	Healthy	200/200	91	59	50	144	39	17	DSM- IV	PCR-RFLP	<0.01
Zhang et al. (52)	China	Southern	Healthy	201/199	32	101	68	42	86	71	CARS	TaqMan	0.099
Zhang et al. (53)	China	Northern	Healthy	1505/1308	630	670	205	618	550	150	DSM-IV	PCR-RFLP	0.104
Zhang et al. (54)	China	Northern	Healthy	68/100	30	18	20	71	17	12	DSM-V/ABC	PCR	<0.01

*DSM-IV, Diagnostic and Statistical Manual of Mental Disorder, Fourth Edition; DSM-V, Diagnostic and Statistical Manual of Mental Disorder, Fifth Edition; ADOS, the Autism Diagnostic Observation Schedule; CARS, Childhood Autism Rating Scale; ABC scoring, Autism Behavior Checklist scoring; ADI-R, the Autism Diagnostic Interview Revised; HWE, Hardy-Weinberg equilibrium; PCR-RFLP, polymerase chain reaction–restriction fragment length polymorphism; PCR, polymerase chain reaction*.

### Minor Allele Prevalence

Five studies (23, 26, 33, 44, 45) deviated from HWE were excluded, leaving 12 studies on Northern Han and 13 studies on Southern Han to be pooled. A high between-study heterogeneity (*I*^2^ = 97.6%, *P* < 0.01) was shown among all the studies and the pooled MAF estimated by a random-effect model was 42% (95% CI: 37–46%). There was also heterogeneity (*I*^2^ = 98.5%, *P* < 0.01) among studies on Northern Han (MAF: 45%, 95% CI: 37–52%), and heterogeneity (*I*^2^ = 91.7%, *P* < 0.01) was found among Southern Han studies (MAF: 39%, 95% CI: 35–43%).

### MTHFR C677T and the Risk of Autism

**Figure 2:**
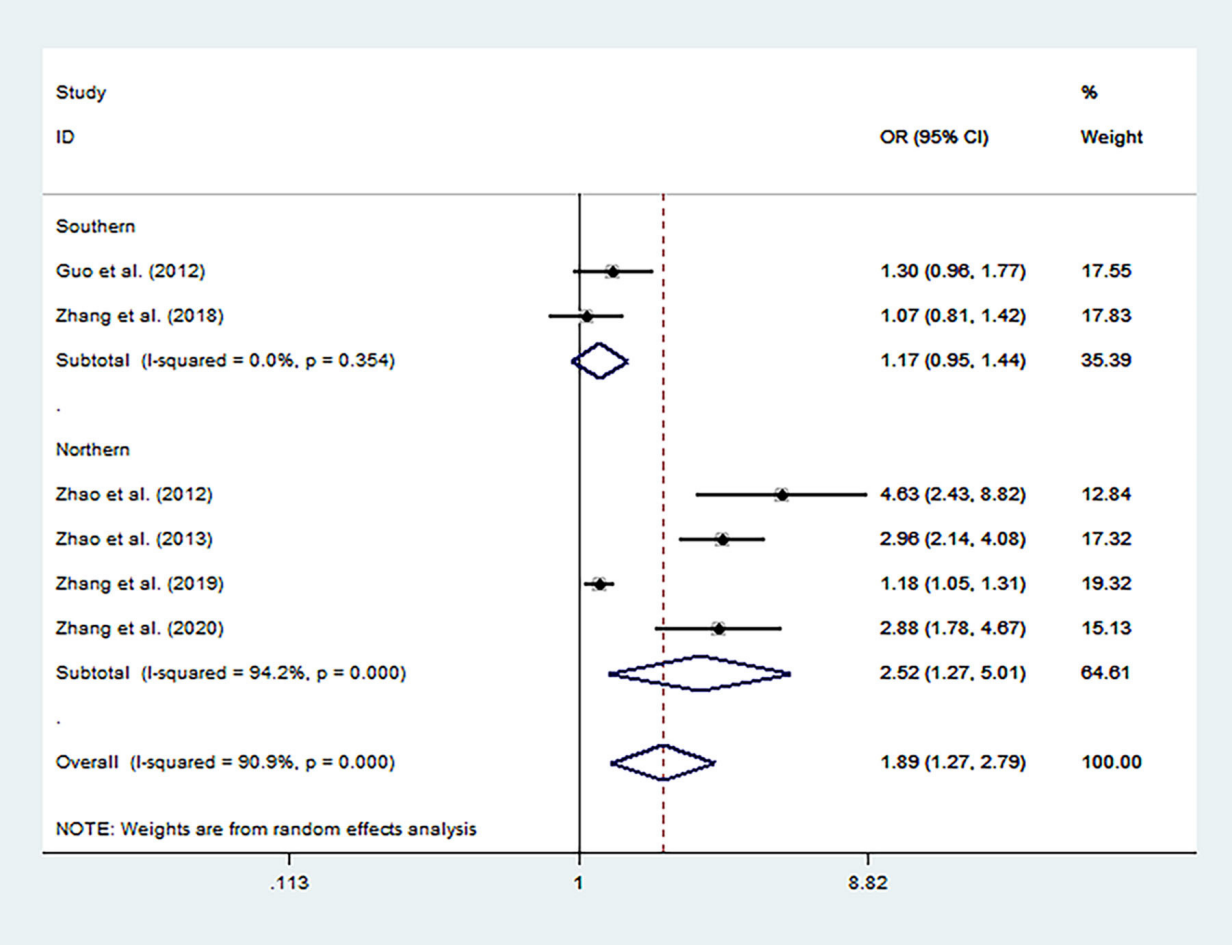
Forest plot for the association between MTHFR C677T polymorphism and autism susceptibility in allele genetic model stratified by region.

Overall analysis showed that the MTHFR C677T polymorphism increased the risk of autism (Table 3) under allele model (T vs. C, OR = 1.89, 95% CI 1.28 to 2.79, Figure 2), homozygous model (TT vs. CC: OR = 2.44, 95% CI 1.43 to 4.15), heterozygous model (CT vs. CC, OR = 1.73; 95% CI 1.19 to 2.51), dominant model (CT + TT vs. CC: OR = 2.03, 95% CI 1.31 to 3.15), and recessive model (TT vs. CT + CC, OR = 1.95, 95% CI 1.21 to 3.13).

**Table 3:** Overall analyses and sensitivity of MTHFR C677T and autism susceptibility.

**Genetic model**	**Subgroup**	**No. of studies**	**Meta-analysis**		***P* for Egger's test**	**Heterogeneity**	
			**OR(95%CI)**	***P*****-value**		***I**^**2**^***(%)**	***P*****-value**
T vs. C	Overall	6	1.89(1.27–2.79)	<0.01	0.08	90.9	<0.01
	HWE-Yes	3	1.18(1.07–1.30)	<0.01		0	0.65
	Northern	4	2.52(1.27–5.01)	<0.01		94.2	<0.01
	Southern	2	1.17(0.95–1.44)	0.13		0	0.354
TT vs. CC	Overall	6	2.44(1.43–4.15)	<0.01	0.07	79.1	<0.01
	HWE-Yes	3	1.38(1.12–1.71)	<0.01		0	0.47
	Northern	4	3.26(1.37–7.76)	<0.01		86.6	<0.01
	Southern	2	1.55(0.96–2.51)	0.07		17.2	0.27
CT vs. CC	Overall	6	1.73(1.19–2.51)	<0.01	0.08	74.1	<0.01
	HWE-Yes	3	1.20(1.04–1.38)	0.01		0	0.51
	Northern	4	2.21(1.19–4.08)	0.01		82.9	<0.01
	Southern	2	1.21(0.82–1.81)	0.34		25.6	0.25
CT+TT vs. CC	Overall	6	2.03(1.31–3.15)	<0.01	0.08	85.5	<0.01
	HWE-Yes	3	1.23(1.08–1.41)	<0.01		0	0.86
	Northern	4	2.67(1.30–5.46)	0.01		91	<0.01
	Southern	2	1.27(0.93–1.75)	0.49		0	0.61
TT vs. CT+CC	Overall	6	1.95(1.21–3.13)	<0.01	0.08	78.7	<0.01
	HWE-Yes	3	1.23(0.88–1.72)	0.22		52.8	0.12
	Northern	4	2.56(1.22–5.40)	<0.01		82.9	<0.01
	Southern	2	1.32(0.61–2.87)	0.04		76	0.04

**Figure 3:**
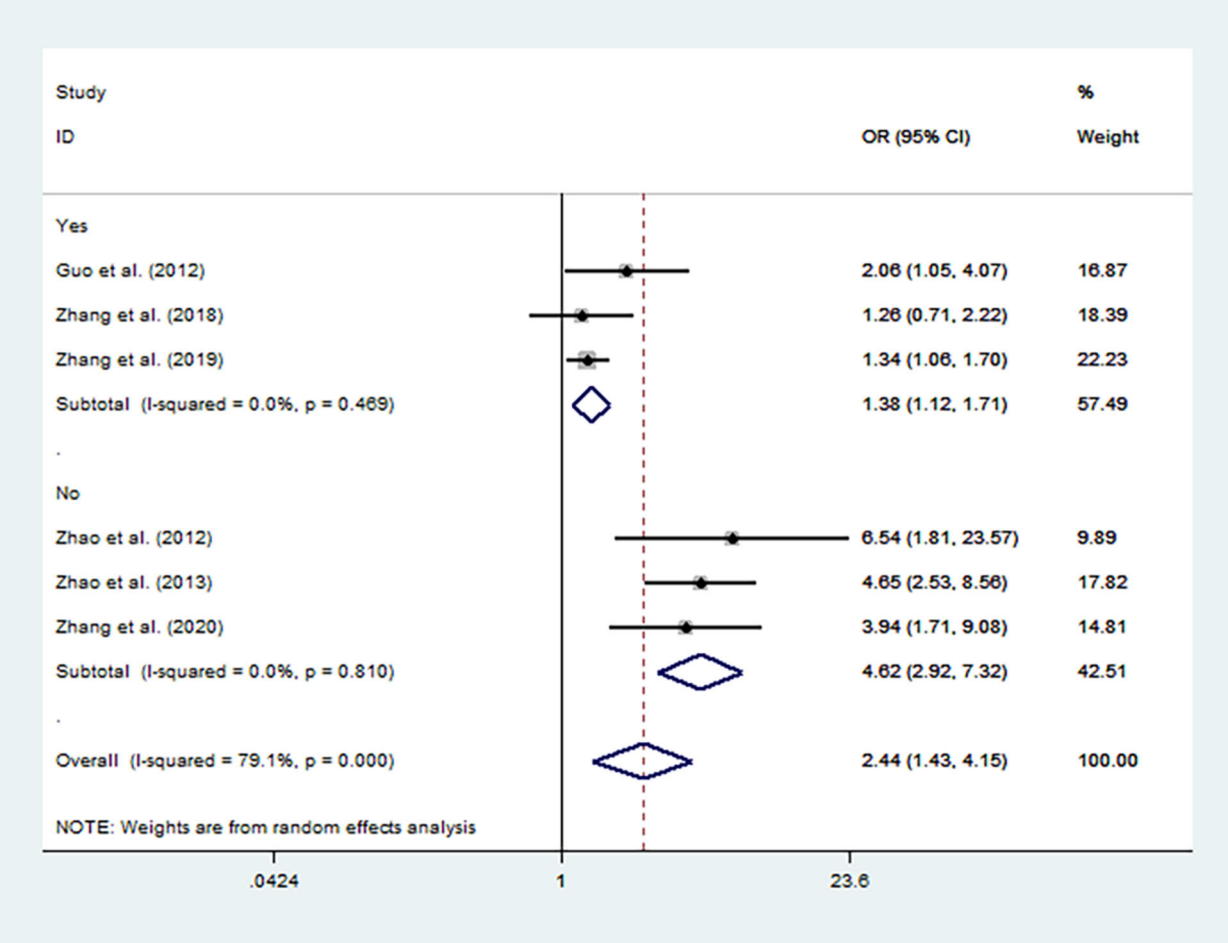
Forest plot for the association between MTHFR C677T polymorphism and autism susceptibility in homozygous genetic model stratified by HWE status of controls.

**Figure 4:**
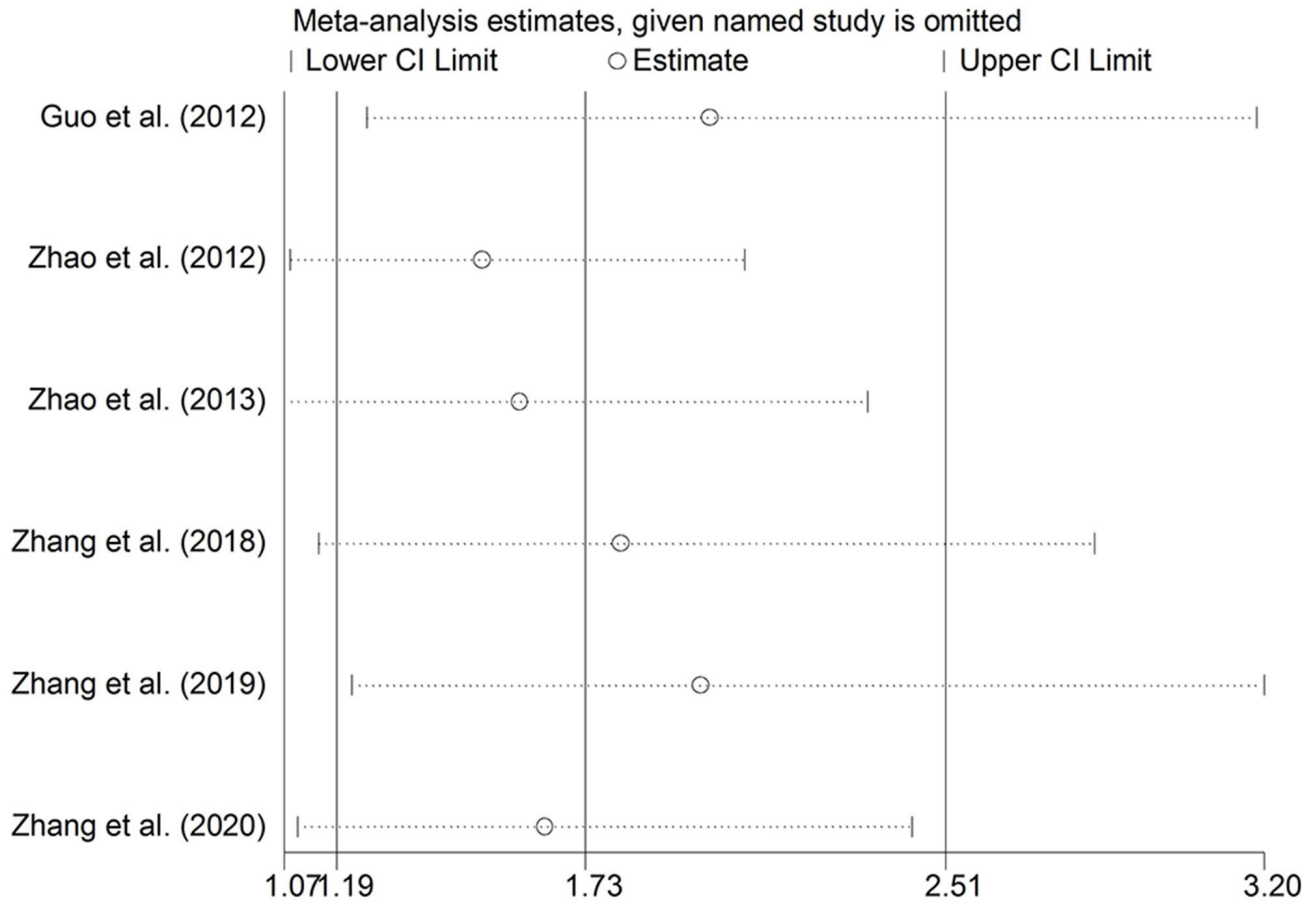
Sensitivity analysis through the deletion of each study to reflect the individual influence on the calculated ORs in a heterozygous genetic model of MTHFR C677T polymorphism.

Sensitivity analysis was performed by removing the studies in which the controls were not consistent with HWE and then the pooled OR for the remaining studies was recalculated. The sensitivity analysis suggested similar patterns to the overall analyses (Figures 3, 4).

### Significant Association of MTHFR C677T Polymorphism and the Risk of Autism in the Northern Han Subgroup

Subgroup analyses showed that when stratified by region, there was a significant association between MTHFR C677T polymorphism and an increased risk of autism in the Northern Han Subgroup under five models (T vs. C: OR = 2.52, 95% CI 1.27 to 5.01; TT vs. CC: OR = 3.26, 95% CI 1.37 to 7.76; CT vs. CC: OR = 2.21; 95%CI 1.19 to 4.08; CT+TT vs. CC: OR = 2.67, 95% CI 1.30 to 5.46; TT vs. CT + CC: OR = 2.56, 95% CI 1.22 to 5.40).

### No Significant Association of MTHFR C677T Polymorphism and the Risk of Autism in the Southern Han Subgroup

By contrast, a significant correlation was absent in the Southern Han subgroup analyses in each model mentioned above (T vs. C: OR = 1.17, 95% CI 0.95 to 1.44; TT vs. CC: OR = 1.55, 95% CI 0.96 to 2.51; CT vs. CC: OR = 1.21; 95%CI 0.82 to 1.81; CT+TT vs. CC: OR = 1.27, 95% CI 0.93 to 1.75; TT vs. CT + CC: OR = 1.32, 95% CI 0.61 to 2.87).

### FPRP Test Results

We drew on an FPRP test to investigate whether the significant associations (*P* < 0.05) detected in the present study were a false positive effect. The results of the FPRP test (see Table 4) indicated that the MTHFR C677T polymorphism was associated overall with autism susceptibility in all gene models. In addition, the FPRP test suggested a truly significant association of MTHFR C677T polymorphism with autism susceptibility in Northern Han instead of Southern Han. These results showed consistent patterns with those reported in the preceding sections.

**Table 4:** The results of FPRP test in each gene model.

**Genetic model**	**Subgroup**	**OR**	**95%CI**	***P*-value**	**Prior probability = 0.25 (FPRP‡ value)**
T vs. C	Overall	1.89	1.27–2.79	0.001	0.007
	HWE-Yes	1.18	1.07–1.30	<0.001	0.002
	Northern	2.52	1.27–5.01	0.008	0.090
	Southern	1.17	0.95–1.44	–	–
TT vs. CC	Overall	2.44	1.43–4.15	<0.001	0.013
	HWE-Yes	1.38	1.12–1.71	0.003	0.010
	Northern	3.26	1.37–7.76	0.008	0.144
	Southern	1.55	0.96–2.51	–	–
CT vs. CC	Overall	1.73	1.19–2.51	0.004	0.015
	HWE-Yes	1.20	1.04–1.38	0.011	0.031
	Northern	2.21	1.19–4.08	0.011	0.083
	Southern	1.21	0.82–1.81	–	–
CT+TT vs. CC	Overall	2.03	1.31–3.15	0.002	0.010
	HWE-Yes	1.23	1.08–1.41	0.003	0.009
	Northern	2.67	1.30–5.46	0.007	0.091
	Southern	1.27	0.93–1.75	–	–
TT vs. CT+CC	Overall	1.95	1.21–3.13	0.006	0.030
	HWE-Yes	1.23	0.88–1.72	–	–
	Northern	2.56	1.22–5.40	0.014	0.136
	Southern	1.32	0.61–2.87	–	–

### Publication Bias

**Figure 5:**
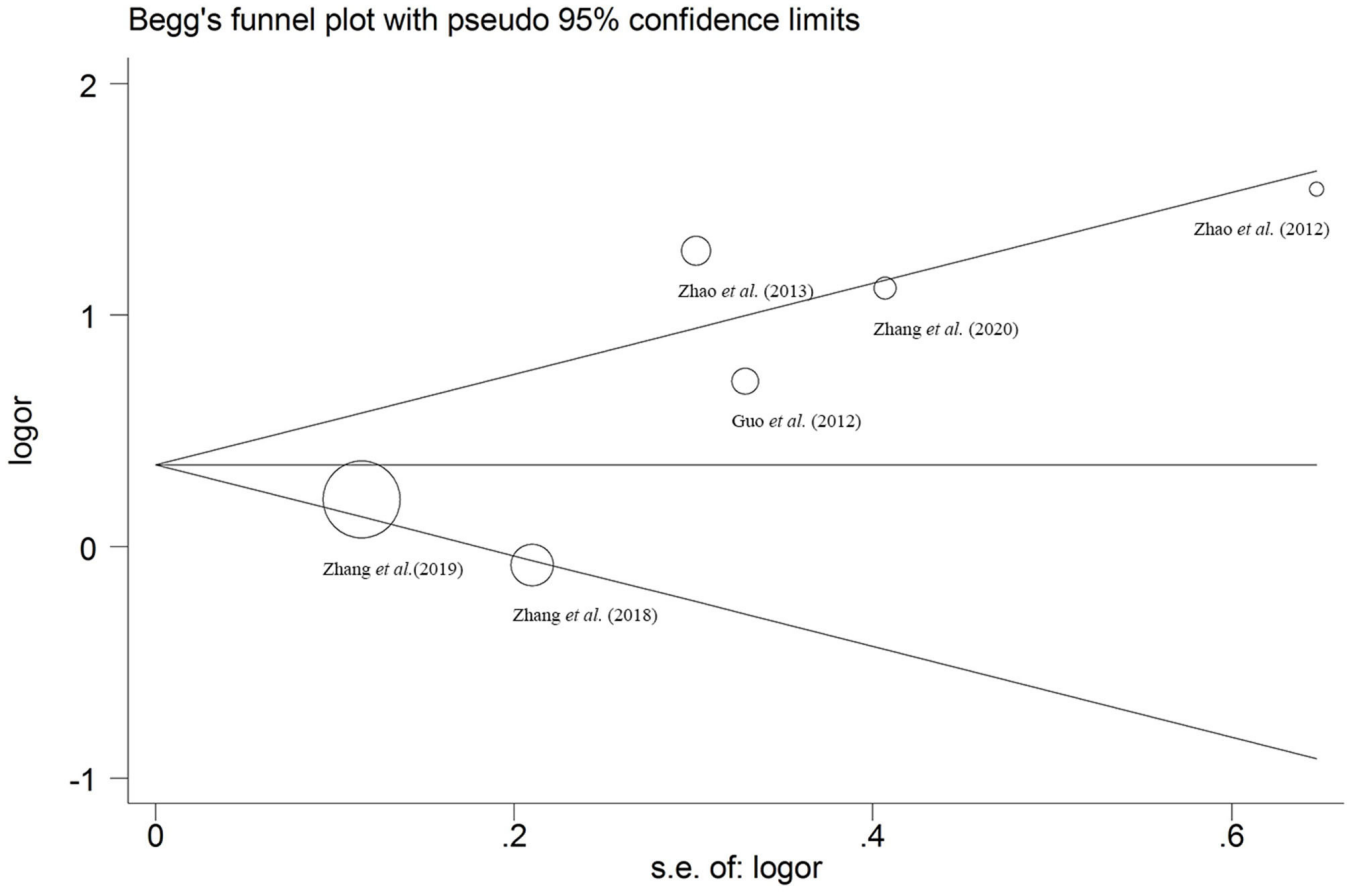
Begg's funnel plot of publication bias for a recessive model of MTHFR C677T polymorphism.

The results of Egger's linear regression tests indicated no significant publication bias (*P* < 0.05), and meanwhile, the Begg's funnel plots were consistent with the conclusion (Figure 5).

## Discussion

To our knowledge, the present meta-analysis is the first to investigate the association between MTHFR C677T polymorphism and the risk of autism in the Chinese Han population. The results suggested that the MTHFR C677T was significantly associated with the increased risk of autism under all genetic models in China, which is in accordance with previous studies. The significant association between the C>T and ASD is consistent with previous results in American (55), Indian (56), and Egyptian (57) populations. Furthermore, a strong relationship between MTHFR C677T and the risk of autism was shown among the Northern Han subgroup, but not the Southern Han subgroup. The above-mentioned results were all consolidated by the FPRP test.

Given that autism is a multifactorial disorder, epigenetic mechanisms play a vital role in the expression of autism phenotypes (58), and are affected by nutritional status as well as medication (59). As a key enzyme of folate metabolism in the process of one-carbon metabolism, the activity of Methylenetetrahydrofolate reductase (MTHFR) strongly affects the one-carbon (C1) metabolic pathway, which is central to cellular methylation reactions. In detail, MTHFR catalyzes the conversion of 5,10-methylenetetrahydrofolate to 5-methyltetrahydrofolate and the latter is required for the conversion of homocysteine to methionine by methionine synthase (60). The MTHFR C677T polymorphism results in a thermolabile variant of MTHFR with a decreased enzyme activity and functions as a well-established genetic determinant of elevated plasma tHcy (total homocysteine; all the circulating forms of Hcy) levels (61). A shift in the glutathione redox ratio and redox imbalance may contribute to the etiology of autism (62). Meanwhile, MTHFR C677T can interact with other SNPs (63–65). For example, the synergistic interactions between MTHFR C677T and MTRR A66G tend to cause an increase in homocysteine, which makes the MTHFR C677T polymorphism a risk factor for autism (66).

In the subgroup analysis, a strong relationship between MTHFR C677T and the risk of autism was shown among the Northern Han subgroup, but not the Southern Han subgroup. These patterns are similar to other studies with a focus on regional subgroup differences in China. These prior studies indicate significant associations between the MTHFR C677T polymorphism and an increased risk of various diseases and disorders, including lung cancer (67), non-syndromic cleft lip and palate (68), depression (69), diabetic nephropathy (70) in the North China population, but such associations were absent (67, 68) or weaker (69, 70) in the South China population. One possible explanation for this may be related to people's different folate and vitamin B12 concentrations in the two regions. A cross-sectional survey conducted by Ren et al. (71) showed that the women in the north had less than half the folate concentration relative to the women in the south. This can be attributed to the fact that the southern region is one of the wealthiest regions in China, and there is a longer growing season with higher temperatures in the south. A survey in 2019 (72) suggested significant differences in distribution characteristics of C677T gene polymorphism of MTHFR between the northern and southern regions, and that Han nationality women in the north had a higher risk of folate in dysmetabolism than the women in the south. Furthermore, Hao et al.'s (73) study on vitamin B12 also showed a similar pattern.

Inspired by these relevant studies, the North-South difference detected in the present meta-analysis may be attributed to the influence of vitamin B12 and folate on the association between the MTHFR C677T and the risk of ASD. Vitamin B12 and folate participate in the methylation cycle as well as in DNA and RNA biosynthesis. Low folate concentrations lead to decreased methylation of proteins, phospholipids, DNA, and neurotransmitters. Al-Batayneh et al. (60) found a significant association between homozygous MTHFR C677T variant as well as T allele frequencies and vitamin B12 deficiency. Through a large-scale study (*N* = 365), Jacques et al. (74) found that when the plasma folate level was lower than 14.5 μmol/l, the plasma Hcy level of the MTHFR gene mutation group was significantly higher than that of the normal genotype group. Therefore, it is suggested that proper levels of vitamin B12 and folate are needed to regulate the metabolism of Hcy in MTHFR gene mutation, to maintain its balance *in vivo*.

Empirically, compensatory folate and vitamin B12 intake can be used to prevent the increase of Hcy level in MTHFR gene mutation. The influences of diet during the periconceptional period are of primary importance for the establishment of DNA methylation patterns and the epigenetic effects caused by these patterns have the potential to persist throughout the life span (75). Oxidative stress may function as a contributing factor to autism pathology. Folate effectively reduced oxidative stress and restored normal concentrations of antioxidant enzymes. Two large-scale case-control studies (76, 77) suggested that the risk for ASD children among the mothers with the MTHFR 677TT genotype is reduced when folate and prenatal vitamin supplements were taken periconceptionally and in the first trimester of pregnancy. According to a mouse study (78), prenatal or early postnatal supplementation of methyl-donors (e.g., folate) decreased the risk of MTHFR-deficiency mice to present ASD-like behavior.

Above all, it is worth paying more attention to genetic screening for women of childbearing age and newborn babies to assess the genetic risk of folate metabolism disorders. On an individual level, genotype/metabolic phenotype analysis tends to guide effective intervention and shed light on the foundations for individual differences in response to treatment (55). A genetic deficiency concerning the MTHFR gene may directly affect metabolite availability and control the environment of the developing embryonic brain in an indirect way (79). The abnormal metabolic profile caused by MTHFR C677T polymorphism can be reduced or counteracted by nutrition treatment (80). Furthermore, treatment for ASD children is effective in correcting metabolic derangements and potentially likely to ameliorate autistic symptoms (81). The intake dosage is also important and of note. According to a study conducted by Raghavan et al. (82), the moderate intake (3–5 times/week) of multivitamin supplements during pregnancy may reduce the risk of ASD in offspring, but very high levels of maternal plasma folate and B12 (≥90%) at birth associated with increased risk of ASD. In other words, both deficient and excessive nutrient status might be associated with an elevated risk of ASD. Proper intake doses of folate and vitamin B12 based on individual needs could not only improve lower tHcy but also avoid the potential adverse effects of excessive intake. Therefore, when the C677T gene polymorphism is detected, targeted nutrition treatment therapies can be expected, which are tailored to individual folate and vitamin B12 levels and genetic background. The above-mentioned measures are likely to reduce the prevalence of autism in China, especially in the Northern Han population.

There are some limitations in the present study. First, as one common limitation in genetic association meta-analysis, heterogeneity may function as a confounding factor in the present meta-analysis. Many factors including experimental design, genetic testing methods, the accuracy of laboratory equipment, etc., may result in heterogeneity (83). To address this issue, a sensitivity analysis was conducted by removing the studies where the controls were not consistent with HWE, with the findings indicating that the results are stable and not significantly constrained by any single study. Second, a single included study having more than half of all participants may bias the current results. Third, the lack of some factors such as oxidant proteins and anti-oxidant status blood tests in the origin articles may influence the conclusion. Finally, publication bias might exist even though no significant publication bias was observed through the Begg test and Egger test. In this regard, more well-designed studies with a large sample size are needed to elucidate the conclusions.

In summary, the present meta-analysis is the first to provide refined current evidence of MTHFR C677T polymorphism with the increased risk of autism in Chinese Han. These results suggest that the MTHFR C677T polymorphism might be a risk factor for ASD in Chinese Han, especially in the north. For those mothers and children who are generally susceptible to autism, tailored nutrition treatment of prenatal folate and vitamin B12 may reduce the risk of having children with autism, especially in the north. Further studies with greater gene-environment statistical power are encouraged to verify our conclusions, taking into account more precise analysis of factors such as age, gender, and lifestyle factors in the development of autism.

## Data Availability Statement

The datasets presented in this study can be found in online repositories. The names of the repository/repositories and accession number(s) can be found in the article/supplementary material.

## Author Contributions

H-FL and C-XL designed the study. C-XL, Y-GL, Y-PC, J-LO, and W-CR performed the literature search, data extraction, and statistical analysis. C-XL drafted the manuscript. Y-GL, Y-LY, and H-FL revised the manuscript. All authors reviewed and approved the final paper for submission and publication.

## Conflict of Interest

The authors declare that the research was conducted in the absence of any commercial or financial relationships that could be construed as a potential conflict of interest.
